# Pembrolizumab-Induced Myocarditis and Delayed Acute Inflammatory Demyelinating Polyradiculoneuropathy

**DOI:** 10.7759/cureus.27112

**Published:** 2022-07-21

**Authors:** Fazal Dalal, Hussain Dalal, Brad Baltz

**Affiliations:** 1 Internal Medicine, Arkansas College of Osteopathic Medicine, Fort Smith, USA; 2 Internal Medicine, Nuvance Health, Poughkeepsie, USA; 3 Department of Hematology and Oncology, Catholic Health Initiatives St. Vincent Infirmary, Little Rock, USA

**Keywords:** immune-mediated injury, immune-checkpoint inhibitors, acute inflammatory demyelinating polyradiculoneuropathy, myocarditis, pembrolizumab

## Abstract

Pembrolizumab is an immunotherapeutic agent used in various malignancies including metastatic melanoma. While immunotherapies are effective in treating several malignancies, they do come at the expense of inadvertent side effects. The numerous side effects of pembrolizumab, including, but not limited to, adrenal insufficiency, myocarditis, and pancreatitis, are well documented in clinical literature. In this case report, we describe a unique presentation of myocarditis and acute inflammatory demyelinating polyradiculoneuropathy secondary to pembrolizumab. While both side effects of pembrolizumab are well known, the delayed presentation of symptoms is of particular interest in our case report. We hope to inform the clinical community on the pharmacokinetics of pembrolizumab causing the delayed onset of symptoms.

## Introduction

Pembrolizumab is a programmed cell death protein-1 (PD-1) inhibitor that binds to its ligand (PD-L1). The expression of PD-L1 is upregulated in many malignancies and has been associated with poor prognosis [1]. By binding to PD-L1, T- lymphocyte proliferation is inhibited, inducing apoptosis of tumor cells [2]. As the use of immunotherapies has been on the rise in treating various malignancies, so has the rise in immune-related adverse effects. In this case report, we present a patient found to have myocarditis and a delayed presentation of acute inflammatory demyelinating polyradiculoneuropathy (AIDPR) after two cycles of pembrolizumab. It has been reported that the terminal half-life of pembrolizumab is approximately 27 days [3]. While there are multiple dosing options, the approved dosage of pembrolizumab is 200 mg intravenously (IV) every three weeks. Although clearance of pembrolizumab depends on many factors such as body weight and renal function, the focus is on the half-life and approximate quantity of the drug causing the side effects [4]. Immunotherapies have been the new innovative answer to malignancies; however, our goal is to educate the medical community about rare serious adverse events associated with immunotherapy.

## Case presentation

**Figure 1:**
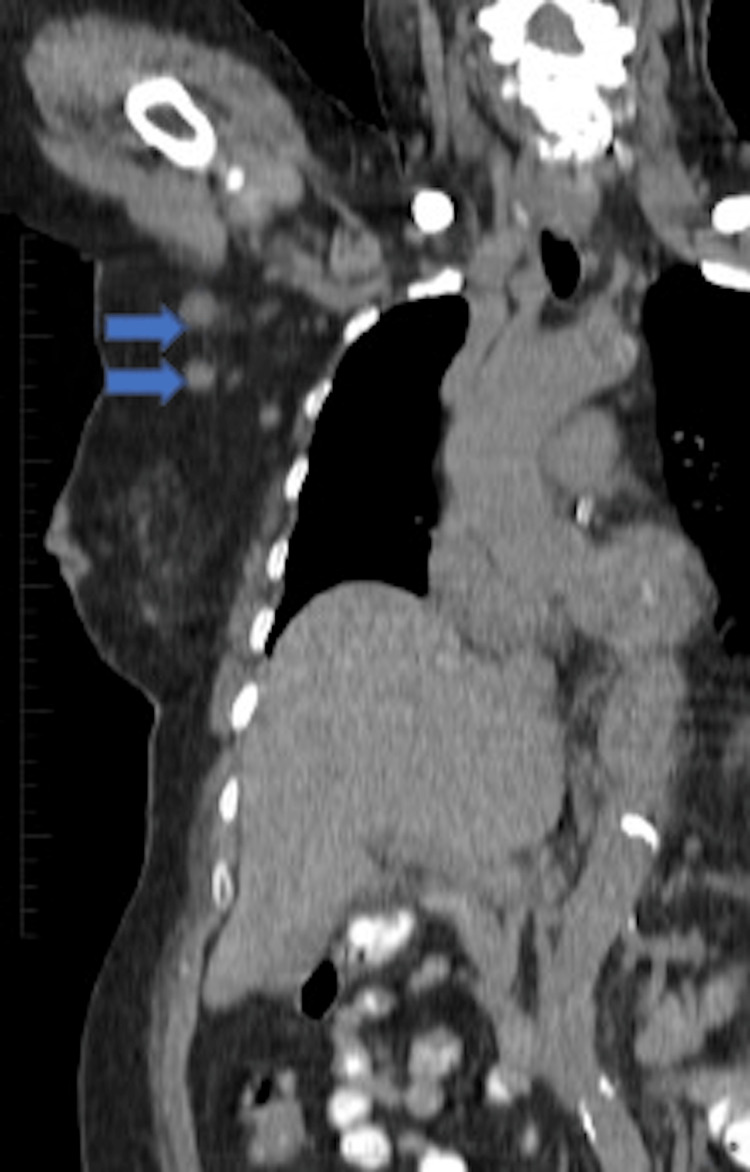
PET-CT in coronal view of the mid-thoracic/axillary region showing approximately 2 cm axillary lymph nodes. PET-CT: positron emission tomography-computed tomography

**Figure 2:**
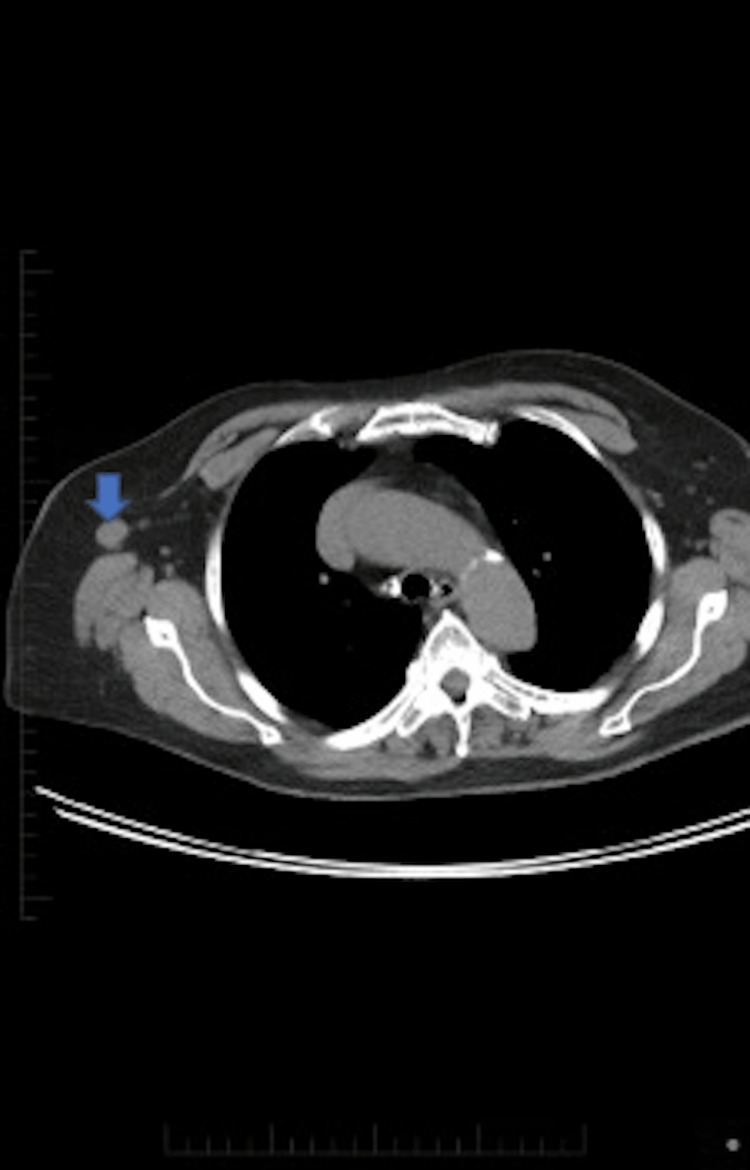
PET-CT with approximately 1.5 cm axillary lymph node. PET-CT: positron emission tomography-computed tomography

**Figure 3:**
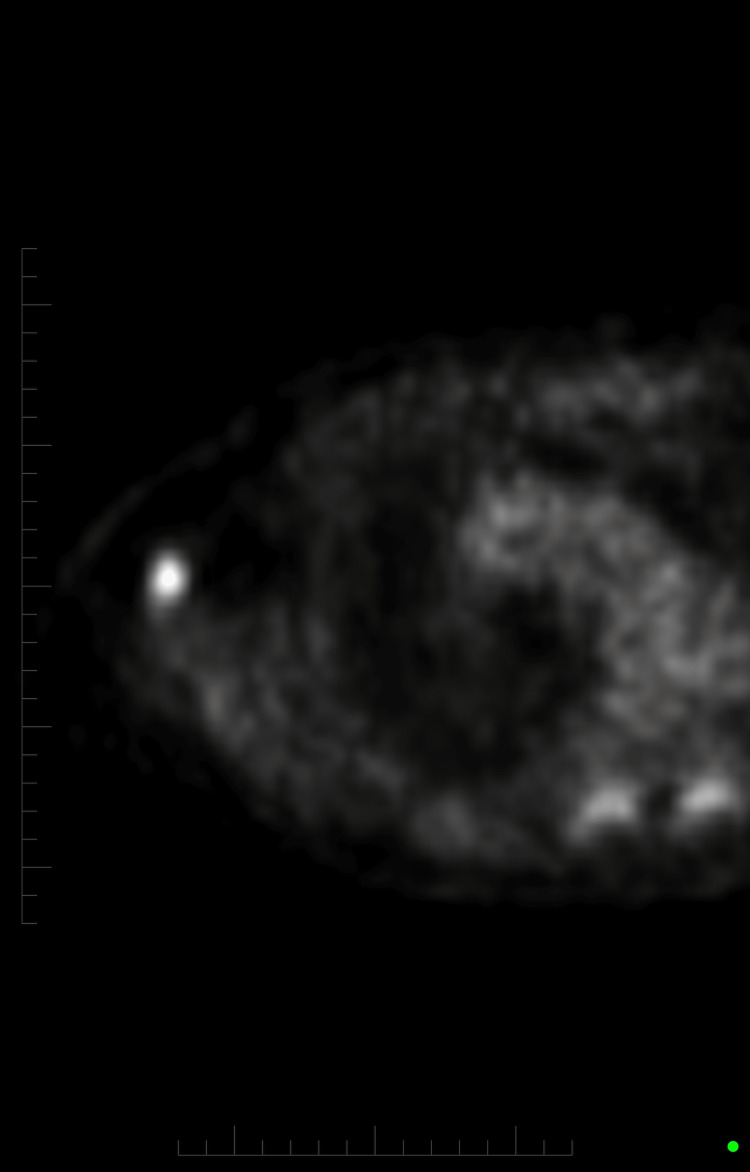
PET-CT showing hypermetabolic process. The radioactive uptake is suggestive of this process confirming metastasis. PET-CT: positron emission tomography-computed tomography

**Figure 4:**
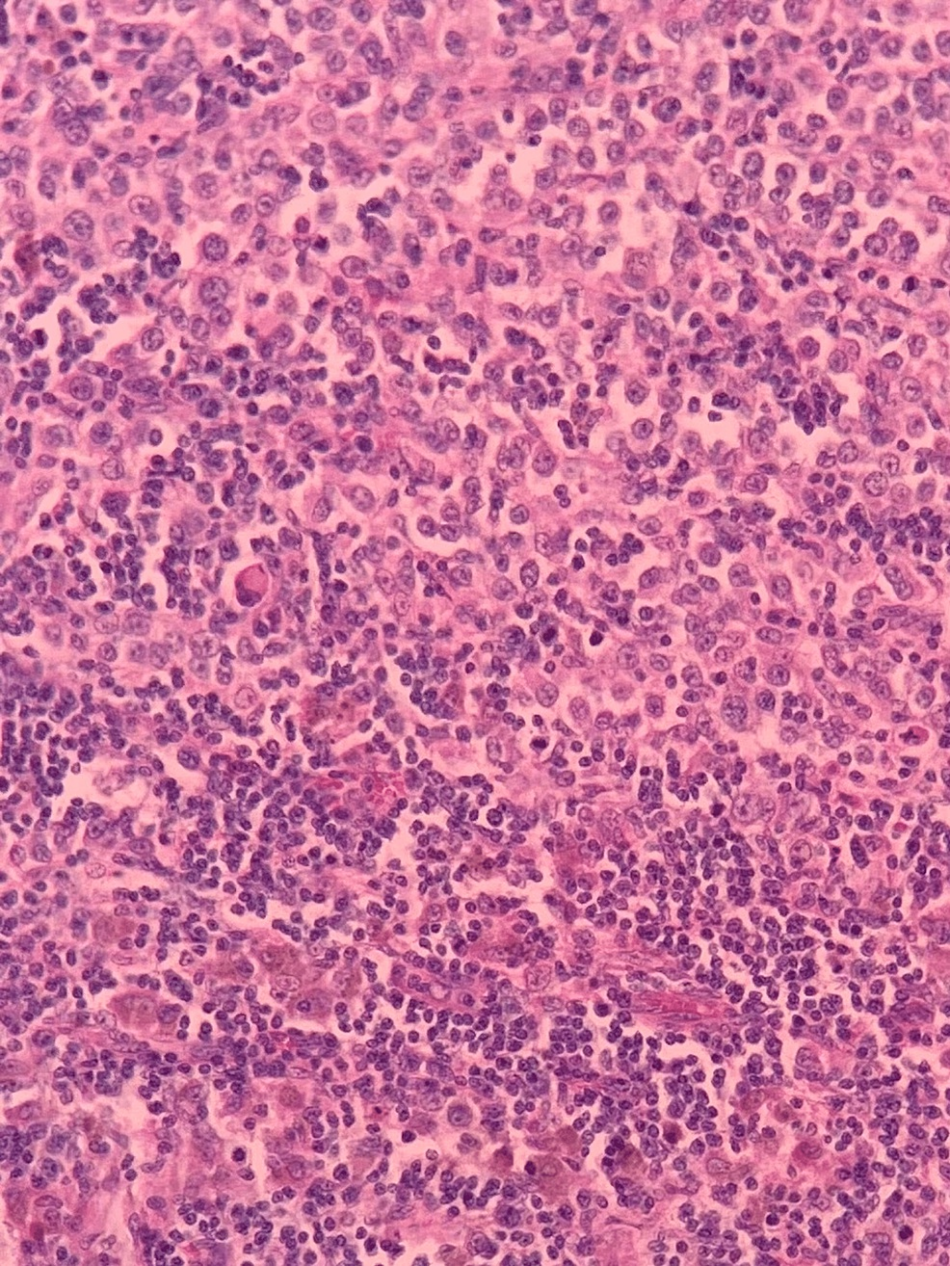
H&E axillary lymph node showing tumor invasion (200×). H&E: hematoxylin and eosin

**Figure 5:**
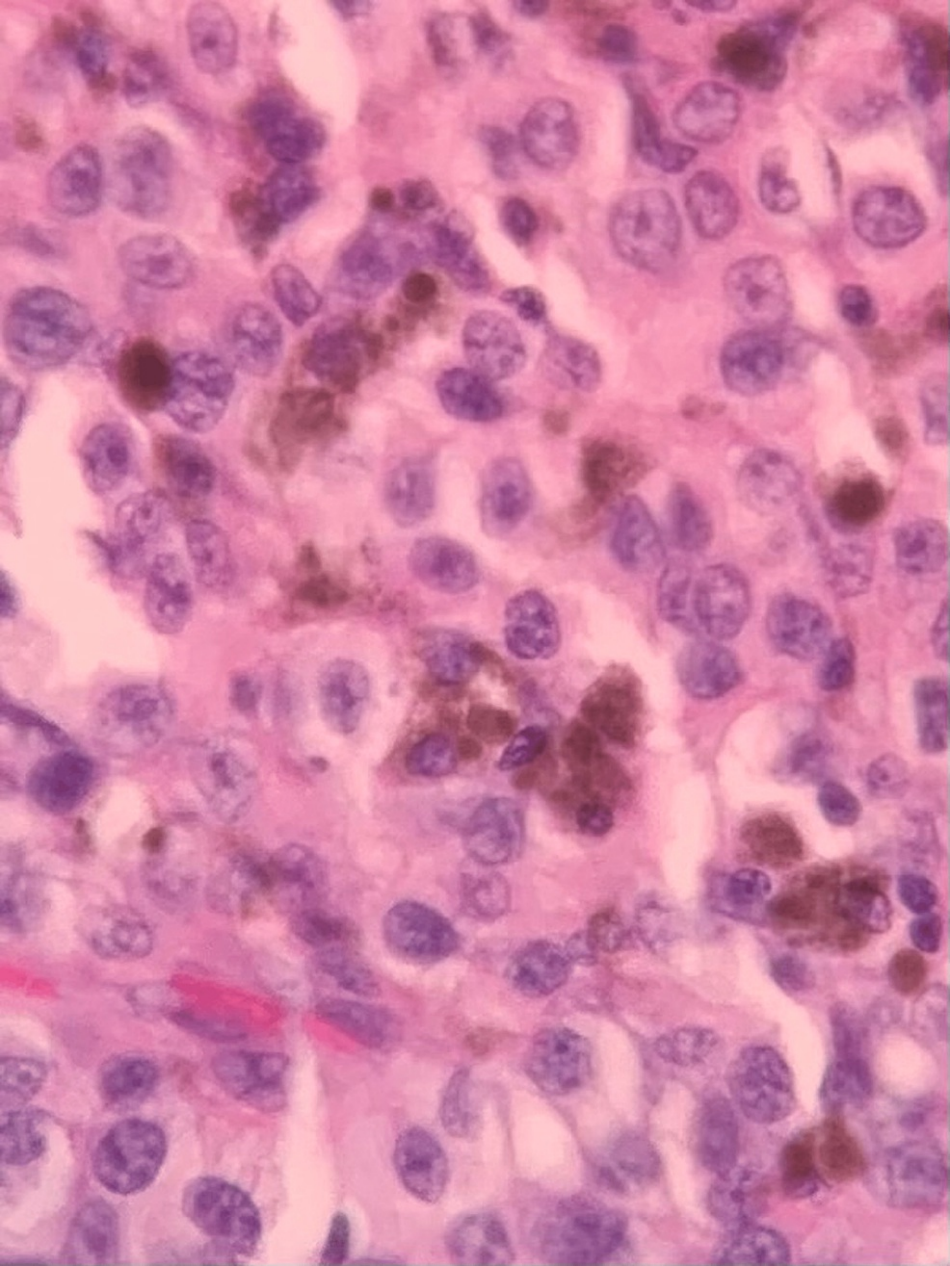
H&E tumor cell with melanin pigment (400×). H&E: hematoxylin and eosin

**Figure 6:**
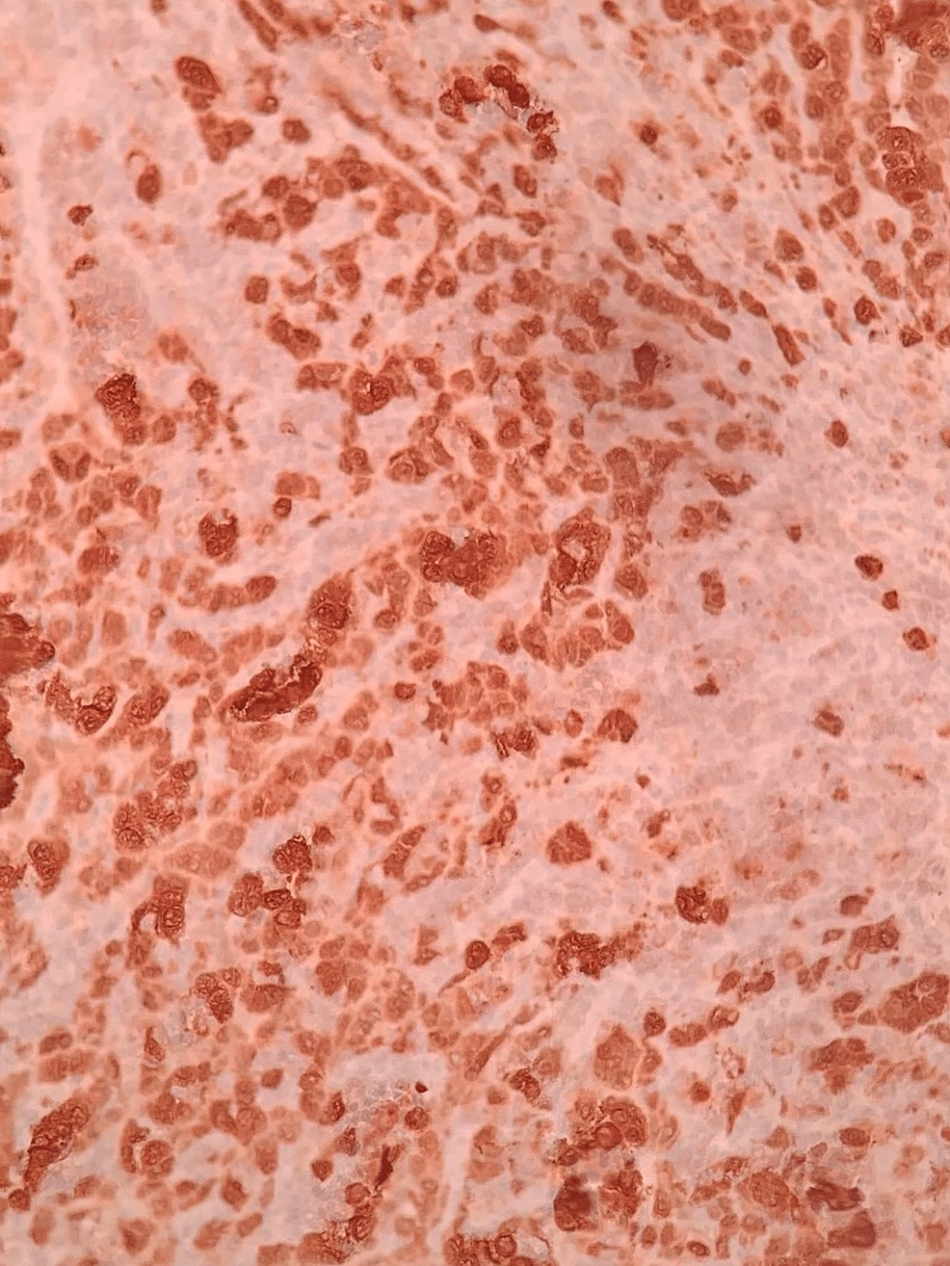
Tumor cell positive for MART-1 stain (400×). MART-1: melanoma antigen recognized by T-cells 1; this is an immunohistochemical marker specific for diagnosing metastatic melanoma.

A 76-year-old female with a medical history of hypothyroidism and iron deficiency anemia presented to her dermatologist with a suspicious right arm nevus. Right proximal posterior upper arm shave resection was done that showed malignant melanoma nodular type with a Breslow thickness of 7.0 mm and Clark level IV. Ulceration was present with cells vocally abutting a deep margin. She was referred to oncology for further assessment and treatment modalities. Positron emission tomography (PET) scan staging was at pT4b (primary tumor at stage 4b) that showed three hypermetabolic right axillary lymph nodes suggesting metastatic melanoma (Figures 1-3). Pathology slides showed invasion of melanoma in the lymph nodes along with melanoma antigen recognized by T-cells 1 (MART-1) staining, a specific immunohistochemical marker for the diagnosis of metastatic melanoma (Figures 4-6). The patient was commenced on a cycle of pembrolizumab 200 mg every three weeks.

**Figure 7:**
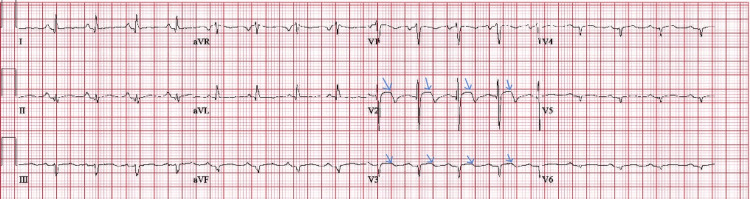
Mild ST-elevations in leads V2/V3 and Q-waves in lateral leads which are often seen in myocarditis after ruling out STEMI from non-obstructive coronary angioplasty. STEMI: ST-elevated myocardial infarction

After the second cycle of pembrolizumab, the patient presented to the emergency room (ER) with fatigue, dyspnea, abdominal pain, nausea, and vomiting. Significant cardiac lab values were noted suggestive of myocardial damage (Table 1). Electrocardiography showed ST-elevations in V2 and V3 (Figure 7). Cardiac catheterization showed non-obstructive coronary arteries and transesophageal echocardiogram showed ejection fraction of >55% with no wall motion abnormality raising suspicion for myocarditis. Due to the unavailability of cardiac magnetic resonance imaging (MRI), the diagnosis was made with these presenting findings. She was commenced on high-dose steroid therapy (1 mg/kg/day) for suspected myocarditis. The following day, she had an episode of excessive vomiting that led to aspiration. The patient went into septic shock along with respiratory failure needing mechanical ventilation; a chest X-ray showed mucus plugging. Therapeutic bronchoscopy was done for mucus washing. The patient’s septic shock worsened. She was started on vasopressor, and prednisone was discontinued after two days of treatment given the underlying infection in the setting of sepsis. After multiple failed ventilator weaning attempts, the patient underwent tracheostomy and percutaneous endoscopic gastrostomy tube placement and was transferred to a long-term acute care facility (LTAC). 

**Table 1:** Cardiac lab values suggesting myocardial damage. CK: creatine kinase; CK-MB: creatine kinase myocardial damage marker; CK index: creatine kinase index

Lab measurement	Lab value	Normal
Troponin I	5.6 ng/mL	<0.045 ng/mL
Total CK	5,590 units/L	25-200 units/L
CK-MB	345.9 ng/mL	0.5-10 ng/mL
CK index	6.2%	2.5-3%

**Figure 8:**
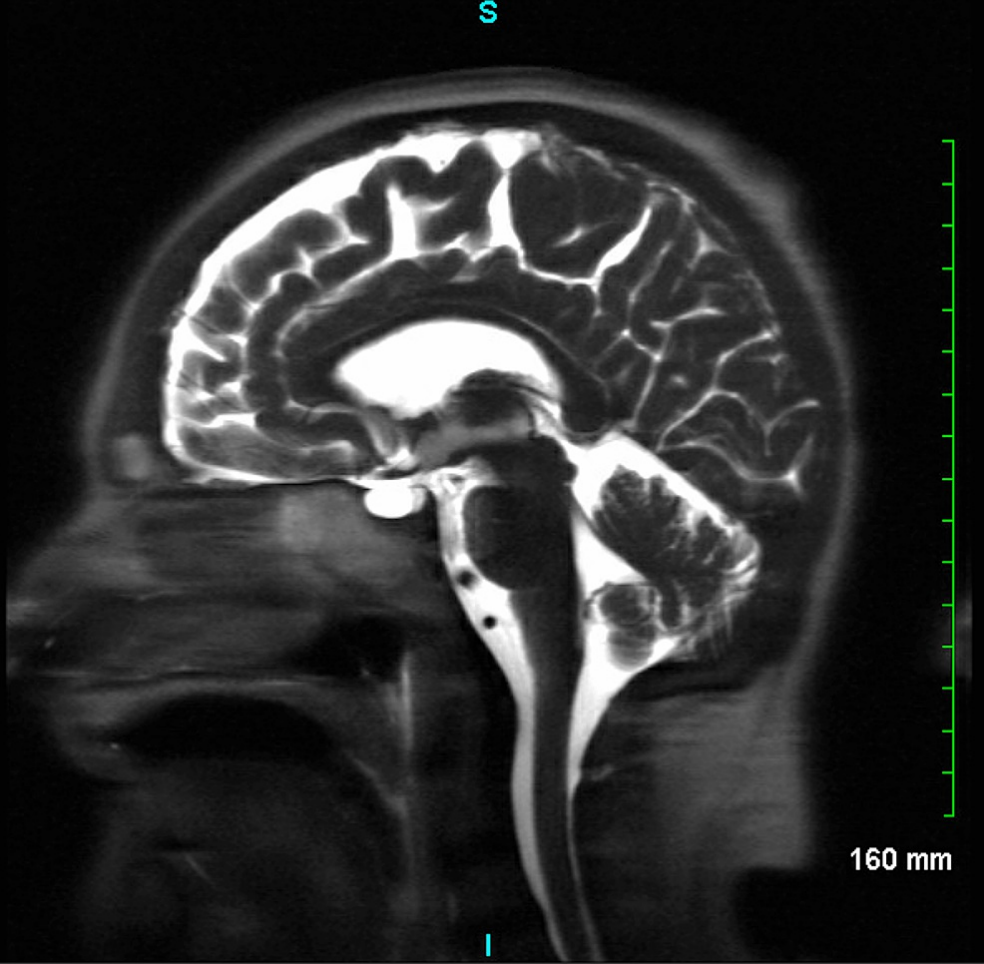
Brain MRI was negative for any findings of acute intracranial processes. White matter abnormalities were consistent with small-vessel ischemic changes and remote bilateral lacunar infarcts in the cerebellum, none of which were pertaining to her symptoms of AIDPR. MRI: magnetic resonance imaging; AIDPR: acute inflammatory demyelinating polyradiculoneuropathy

At LTAC four weeks later, she was found to have apraxia, partial left eye ptosis, weakness of upper and lower extremities, and areflexia prompting an urgent MRI (Figure 8). MRI was unremarkable; however, given her persistent neurological deficits, the patient was readmitted to our institution for further workup. Myasthenia gravis was ruled out after negative acetylcholine receptor (AchR) antibodies on two separate occasions, as well as negative muscle-specific kinase (MuSK) antibody and a normal MRI of the orbit. A paraneoplastic panel including ganglioside antibodies and Gq1B antibodies was negative. Lumbar puncture showed albuminocytologic dissociation with cerebrospinal fluid (CSF) proteins at 88 which prompted a working diagnosis of AIDPR. Plasmapheresis and intravenous immunoglobulin were initiated and the patient was successfully weaned to the Passy Muir valve after the seventh cycle of plasmapheresis.

As per protocol, a repeat PET-CT is done after four cycles of pembrolizumab treatment. Because our patient only had two cycles which were ceased after these side effects, we were unable to get a PET-CT to stage after treatment. Unfortunately, our patient passed away a month after discharge with a suspected cardiac arrest. She was found unresponsive in her bed, and with subsequent unsuccessful attempts to revive her in the ER, she succumbed.

## Discussion

Pembrolizumab and other immunotherapies are relatively safe treatment options for melanoma and other solid tumors. The most common side effects are usually cutaneous including skin rashes and pruritus [5]. Rare side effect occurrence is less than 1%, as reported by drug manufacturers, such as cardiac, neurological, hematological, and musculoskeletal. Pembrolizumab was the preferred treatment option in patients such as ours with metastatic melanoma and usually has a very tolerable side effect profile.

There have been several cases of pembrolizumab-induced myocarditis and pembrolizumab-induced AIDPR in patients that have led to discontinuation of the drug; however, none were reported simultaneously in the same individual. Analysis by Robert et al. on the long-term safety of pembrolizumab showed that 17.7% of patients had mild/moderate side effects out of a pool of 1,567 patients that resulted in only two deaths [6]. This study allowed pembrolizumab to be used by oncologists safely as monotherapy in patients with advanced diseases with which side effects can be managed. Pembrolizumab can also be used in patients with failure of ipilimumab treatment and *BRAF* mutations, as approved by the Food and Drug Administration [7].

The delayed presentation of this patient’s neurological symptoms is owed to the pharmacokinetics of pembrolizumab. As per dosing guidelines, pembrolizumab is usually given as a 200 mg IV single dose on day one of a 21-day cycle. Once administered intravenously, the drug is immediately and completely bioavailable and is not expected to bind to plasma proteins [8]. The clearance process of the drug is slow, and the half-life is usually 27 days. With the three-week dosing, the steady-state concentration of the drug is reached by 19 weeks [8]. The time frame between when our patient was first diagnosed with myocarditis and then AIDPR closely coincides with the time it takes for the medication to reach a steady state and cause any potential side effects.

Our case highlights the importance of pharmacokinetics of the drug which can cause the delayed presentation of side effects. While there has been a case reported of delayed acute hepatitis after ceasing pembrolizumab therapy, not many have been reported with multiple side effects, as in our case [9]. Such instances require physicians to be aware of delayed presentations of therapies and appropriately treat them promptly. Our goal is to highlight such rare side effects that can occur in a delayed fashion with pembrolizumab and to alert the medical community on how to approach such cases as they encounter in their clinical practices.

## Conclusions

In summary, we present a unique case of myocarditis and AIDPR with pembrolizumab use for metastatic melanoma after only two cycles. Pembrolizumab is a widely used immunotherapy not only in melanomas but in other solid tumors as well. It has been a great advent in the field of oncology; however, the side effects can be detrimental. As we hope to educate the medical community, clinicians must be aware of such rare side effects of potent immunotherapies and the variety of organ systems they can affect.

## References

[1] Najjar YG, Kirkwood JM (2015). Pembrolizumab: pharmacology and therapeutics review. Am J Hematol Oncol.

[2] de Maleissye MF, Nicolas G, Saiag P (2016). Pembrolizumab-induced demyelinating polyradiculoneuropathy. N Engl J Med.

[3] Li H, Yu J, Liu C (2017). Time dependent pharmacokinetics of pembrolizumab in patients with solid tumor and its correlation with best overall response. J Pharmacokinet Pharmacodyn.

[4] Ahamadi M, Freshwater T, Prohn M (2017). Model-based characterization of the pharmacokinetics of pembrolizumab: a humanized anti-PD-1 monoclonal antibody in advanced solid tumors. CPT Pharmacometrics Syst Pharmacol.

[5] Wang M, Ma X, Guo L, Xia F (2017). Safety and efficacy profile of pembrolizumab in solid cancer: pooled reanalysis based on randomized controlled trials. Drug Des Devel Ther.

[6] Robert C, Hwu WJ, Hamid O (2021). Long-term safety of pembrolizumab monotherapy and relationship with clinical outcome: a landmark analysis in patients with advanced melanoma. Eur J Cancer.

[7] Simeone E, Grimaldi AM, Festino L (2017). Correlation between previous treatment with BRAF inhibitors and clinical response to pembrolizumab in patients with advanced melanoma. Oncoimmunology.

[8] Longoria TC, Tewari KS (2016). Evaluation of the pharmacokinetics and metabolism of pembrolizumab in the treatment of melanoma. Expert Opin Drug Metab Toxicol.

[9] Phan T, Patwala K, Lipton L, Knight V, Aga A, Pianko S (2021). Very delayed acute hepatitis after pembrolizumab therapy for advanced malignancy: how long should we watch?. Curr Oncol.

